# Recovery of Crocins From Floral Tissue of *Gardenia jasminoides* Ellis

**DOI:** 10.3389/fnut.2020.00106

**Published:** 2020-09-02

**Authors:** Sarana Rose Sommano, Pongsakorn Suppakittpaisarn, Korawan Sringarm, Taepin Junmahasathien, Warintorn Ruksiriwanich

**Affiliations:** ^1^Plant Bioactive Compound Laboratory (BAC), Department of Plant and Soil Sciences, Faculty of Agriculture, Chiang Mai University, Chiang Mai, Thailand; ^2^Cluster of Research and Development of Pharmaceutical and Natural Products Innovation for Human or Animal, Chiang Mai University, Chiang Mai, Thailand; ^3^Landscape Design and Environmental Management Studio, Faculty of Agriculture, Chiang Mai, Thailand; ^4^Department of Animal and Aquatic Sciences, Faculty of Agriculture, Chiang Mai University, Chiang Mai, Thailand; ^5^Department of Pharmaceutical Science, Faculty of Pharmacy, Chiang Mai University, Chiang Mai, Thailand

**Keywords:** cape jasmine, carotenoids, chromatography, dye, edible flower, natural pigment

## Abstract

In this research, a novel source of phytopigment crocins from fully open mature flowers of cape jasmine (*Gardenia jasminoides*) is introduced. Methanol and deionized water were appropriate solvents for pigment recovery with maximum yields of at least 17% from the floral tissue. Pigment separation by thin layer chromatography also confirmed the presence of the carotenoids, which dissolved well in these high-strength polar solvents, in fruit, flower, and leaf materials. The spectral patterns of the extracts from ultraviolet and nuclear magnetic resonance showed maximum absorption at ~420 nm and the chemical shift values were similar to those of crocetin aglycones (crocins) in the methanol extracts of a commercial source of yellow gardenia (fructus or fruit of *Gardenia florida*). Chemical compositions were then evaluated using aqueous-phase capillary electrophoresis of the methanol extracts. The methanolic extracts of the flowers and fruit had 11 principal ingredients in common. Among these, crocetin and crocin 2 belong to the crocin group and are known to be the major components of commercial yellow *Gardenia*. This research not only demonstrates a sustainable means of raw material utilization for natural product recovery, but also encourages a movement toward an edible landscape for the community.

**Graphical Abstract:**
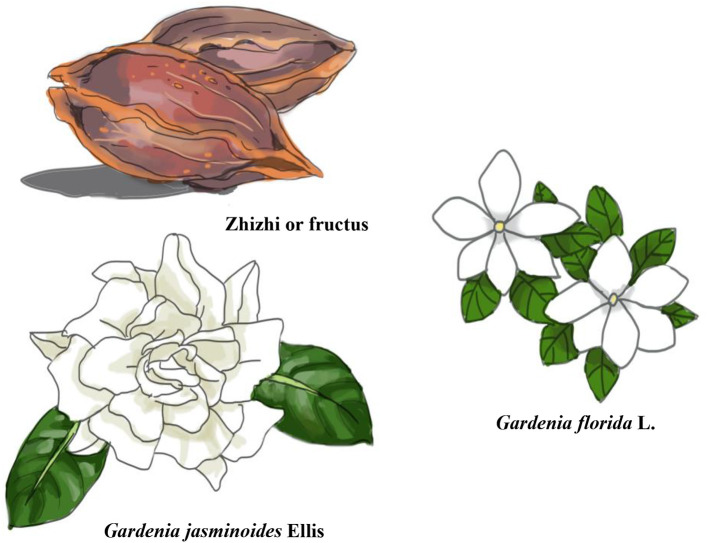
Sources of crocins from *Gardenia* spp.

## Introduction

Natural dye extracts have been mainly used as colorants, additives, or antioxidants for the food, pharmaceutical, and textile industries for almost two centuries. However, the development of synthetic chemical dyes has brought about almost complete substitution of natural products owing to their durable and functional properties, such as color stability, time efficiency, and convenience (1). Fortunately, the “green” movement has been working against the synthesized dye industry because of the negative impacts on the environment of synthetic additives as well as the adverse effects for humans, e.g., toxic or allergic reactions (2). As a result, ecological and economic restrictions on the toxic chemicals, including bans on certain products containing synthetic dyes, have been established (3). Natural dyes are biodegradable and generally environmentally friendly. They also possess potential properties including antioxidant and antimicrobial activities for commercial applications such as food additives, cosmetics, and pharmaceuticals (4–6). Furthermore, the connection between local resources and humans has been challenged through the widespread use of chemical dyes that encourage the need for conservation of local species (7).

*Gardenia* is an evergreen shrub used mostly for landscape functions and is known for its sweet, subtly fragrant flowers. A hybrid variety of this plant, i.e., *Gardenia jasminoides* Ellis or cape jasmine, has been generally used in Thailand for aromatherapy, hair decoration, and religious services (8). There is also a record of the use of the flowers for culinary purposes in Asian countries (9, 10). The fruit of *Gardenia* (fructus or Zhizhi) (*G. florida* L.) is well-recognized in Traditional Chinese Medicine because of its bitter flavor and cold properties. As a result, a medicinal formula containing fructus is used to relieve hepatic and abdominal pains with underlying anti-phlogistic, diuretic, laxative, choleretic, and homeostatic properties (11). The fruit is also a major source of yellow coloring as it is a typical plant carotenoid, the major constituents of which are crocin and crocetin (12, 13). The others are blue genipin-derived pigments from reaction with the amine groups (14). The pigments from this well-known *Gardenia* are utilized as food additives, medicines, and, more recently, natural photosensitizers for dye-sensitized solar cells (15–17) and in the textile industry (3, 17, 18). Besides the extensive use of the fruits as natural pigments, the leaf and floral tissues are also known to have appealing pigments. It has been observed that white, fully open flowers of *Gardenia* turn an appealing yellow after they reach maturity (19). Moreover, it is generally understood that, because of the complex floral structure of *G. jasminoides*, natural fruit setting of this common landscape plant is rarely possible (J. F. Maxwell, personal communication).

Despite the above-mentioned studies, there is a lack of information on the chemical characteristics of yellow *Gardenia* from the floral tissue. This study was, therefore, conducted to characterize the pigments from a substitute raw material source that could replace the use of the propagating materials, i.e., the fruit. The outcomes of this study could also become the platforms for the development of novel environmentally friendly additive products for functional food and textile industries as well as encourage the movement toward a sustainably edible landscape.

## Materials and Methods

### Plant Materials

Inflorescences of the leaves and flowers of the landscape gardenia (*G. jasminoides*) were harvested from the botanical collection garden at the Faculty of Agriculture, Chiang Mai University, Thailand. Flowers at their mature stages (yellowish-white, fully open) and leaves (dark green) were selected (**Figures 2B1,B2**). *Gardenia* fructus (*G. florida*) were obtained from a local farm located in Mea Ei District, Chiang Mai, Thailand. They were pest free, clean, ca. 2–3 cm in diameter, 5–6 cm long, and yellow-orange in color (**Figure 2A**). All materials were dried at 60°C in a hot-air oven for 48 h and stored at room temperature (25°C) in a room without direct sunlight until used. Moisture, fat, and ash contents were analyzed according to AOAC official methods of analysis (20). Plant species were confirmed by a botanical specialist (Dr. J. F. Maxwell, Department of Biology, Faculty of Science, Chiang Mai University, Thailand) using specimens collected from the field. The specimens were also deposited at the herbarium belonging to the Plant Bioactive Compound Laboratory (BACH), Faculty of Agriculture for future reference with voucher record numbers BACH151 and BACH152 for those belonging to *G. jasminoides* and *G. florida*, respectively.

### Thin-Layer Chromatography

Dried tissues (5.0 g each) were ground to fine particles and soaked in 200 mL of each of the following solvents: deionized water (DIH_2_O), dichloromethane, ethyl acetate, and methanol for 48 h. The debris from the plant materials was removed and the extract was evaporated to dryness under vacuum. The yield (%) of the extracts was then obtained. To adjust the concentration, dried extract (0.5 mg) was redissolved in 100 μL of DIH_2_O, chloroform, ethyl acetate, and methanol. Aliquots (5 μL) of the extracts were spotted onto silica gel thin-layer chromatography (TLC) plates (Merk-60 F254, 0.25 mm), dried, and developed in TLC tanks with different types of solvent systems, such polar, medium polar, and non-polar, to separate the polar and non-polar components from the plant materials (21). The mobile phases were (i) benzene–ethyl acetate (18:2) and (ii) chloroform–methanol (19:5).

### Ultraviolet and Nuclear Magnetic Resonance Spectra

The absorption spectra of the extracts were evaluated with wavelengths in the range 200–700 nm by means of an ultraviolet (UV)-visible spectrophotometer (SPECTROstar Nano; BMG LABTECH, Offenburg, Germany) (22, 23). For, ^1^H nuclear magnetic resonance (NMR) spectra, the extracts (1 mg) were dissolved in deuterated dimethyl sulfoxide (DMSO-d6)/deuterium oxide (D_2_O) and the spectral data were recorded on an Avance 300 MHz NMR spectrometer (Bruker, Rheinstetten, Germany). Structural elucidation of the pigments was accomplished by comparison of the ^1^H NMR spectra as described by Choi et al. (22) and Sobolev et al. (24).

### Capillary Electrophoresis–Mass Spectrometry

For an aqueous system, the background electrolyte consisted of 10 mM ammonium acetate, titrated to pH 4.75 with acetic acid, and a constant positive voltage of +30 kV was applied for separation. The sheath–liquid interface was supplied with a 1:1 mixture of 2-propanol with DIH_2_O (resistance >18 MΩ) and 1% acetic acid at a rate of 4 μL/min. Capillary electrophoresis–mass spectrometry (CE-MS) was performed using a 7100 Capillary electrophoresis system (Agilent Technologies, Waldbronn, Germany) coupled with quadrupole time-of-flight–mass spectrometry (qToF-MS) and a 6340 Ion Trap (Agilent Technologies, Santa Clara, CA, USA). A bare fused silica capillary (ø 50 μm and length 67.5 cm) was flushed step-wise with the prepared buffers according to the protocol described by Posch et al. (25). Prior to sample injection, the extracts were diluted (1:100) with methanol and injected hydrodynamically for 10 s at 5 kPa. Only the alcoholic extract was chosen for this test as it is has been proven to be effective for the extraction of crocins and other metabolites from the fructus (13, 26–28). The nebulizer was set to 13.8 kPa during injection and vial handling to prevent air bubbles forming; this was subsequently increased to the desired value of 41.4 kPa at the beginning of voltage application. Instrumental settings for the qToF instrument were as described in Posch et al. (25, 29). Tentative identification of the chemical compounds was performed by using their accurate mass from the qToF–MS and the Sequential product-ion spectra (MS^3^) from the ion trap.

### Statistical and Chemometric Analyses

Analyses of the chemical compositions and yields were done in triplicate and reported as the mean ± standard error. Differences between samples were determined by Duncan's multiple range tests in SPSS statistical program v.17 (SPSS Inc., Chicago, IL, USA). Principal component analysis (PCA) was conducted to describe and interpret the interdependence of the data using the 2018 trial version of XLSTAT (New York, NY, USA). A Venn plot was generated with BioVinci software (BioTuring Inc., San Diego, CA, USA).

## Results and Discussion

### Chemical Compositions and Solvent Extraction of Yellow Dye Additives

Table 1 illustrates the moisture, fat, and ash contents of the leaves, flowers, and fruit of *Gardenia* spp. The fructus had a fat content as high as 17%, followed by the floral and leaf tissues, respectively. Siwawej and Jarayapun (16) reported that the average fat content of *Gardenia* fruit without the pericarp was around 20–30%. The ash content was, however, higher in the leaves, followed by fruit and flower tissues (~7%). The value falls within the reference range of ash content in *Gardenia* fruit (30). Phytopigments are mainly classified into fat-soluble pigments, which are mainly present in the plastid of the plant protoplasm, and water-soluble pigments, which dissolve in the cell sap (6). The fat-soluble pigments are, for instance, chlorophylls and carotenoids and the water-soluble pigments include those belonging to the flavonoids, such as anthoxanthins and anthocyanins (14, 31, 32). It has been generally described that the analyzed fat and ash contents correlate well with the color intensity of both fat- and water-soluble pigments, respectively. Siwawej and Jarayapun (16) described that the lower fat content of *Gardenia* fruit corresponded to high color absorption intensity. In wheat flour, the yellow pigments were also shown to have a strong relation with ash content (33).

**Table 1:** Chemical compositions of different parts of *Gardenia* spp.

**Gardenia parts**	**Moisture content (%)**	**Fat content (%)**	**Ash content (%)**
Leaf^*^	95.83 ± 0.06^b^	7.2 ± 0.021^a^	7.34 ± 0.05^c^
Flower^*^	95.69 ± 0.06^b^	10.90 ± 0.17^b^	5.92 ± 0.11^a^
Fruit^**^	92.04 ± 0.03^a^	17.50 ± 0.09^c^	6.95 ± 0.03^b^

Values are the mean of triplicate samples ± standard error. Within the same column, values with the same superscript letter (a–c) are not significantly different at p = 0.05.

* The sample was botanically described as belonging to G. jasminoides (BACH151).

** *The sample was botanically described as belonging to G. florida (BACH152)*.

### Organic Solvent Extraction and Thin Layer Chromatography

Different solvents were used to extract natural yellow dyes from different *Gardenia* parts (Table 2). Among the solvent combinations tested, methanol gave the highest yield followed by DIH_2_O, ethyl acetate, and dichloromethane. Kesavan et al. (30) reported a low yield of *Gardenia* extracts when low polar organic solvents (namely, ethyl acetate and dichloromethane) were used. The yellow pigment appeared in high polar organic solvents (methanol, DIH_2_O, and ethyl acetate), mainly from the fruit and floral tissues of *Gardenia*. Methanol and DIH_2_O extracts of leaf tissue were green, whereas ethyl acetate extracts of leaf and flower tissues and dichloromethane extracts were black (Table 2).

**Table 2:** Yield (%) and color description of the *Gardenia* spp. extracts from different parts of the plant.

**Type of extract**	***Gardenia* spp. part**	**Yield (%)**	**Pigment description**
Methanol	Leaf^*^	26.24 ± 0.11^a^	Green
	Flower^*^	15.91 ± 0.10^e^	Yellowish-brown
	Fruit^**^	12.98 ± 0.24^f^	Yellow
Ethyl acetate	Leaf^*^	1.67 ± 0.34^j^	Black
	Flower^*^	3.61 ± 0.56^i^	Black
	Fruit^**^	17.35 ± 0.13^d^	Yellow
DIH_2_O	Leaf^*^	19.80 ± 0.10^c^	Green
	Flower^*^	17.24 ± 0.21^d^	Yellowish-brown
	Fruit^**^	23.01 ± 0.15^b^	Yellow
Dichloromethane	Leaf^*^	9.96 ± 0.12^g^	Black
	Flower^*^	7.21 ± 0.32^h^	Black
	Fruit^**^	6.76 ± 0.28^h^	Black

Values are the mean of triplicate samples ± standard error. Within the same column, values with the same superscript letter (a–j) are not significantly different at p = 0.05.

* The sample was botanically described as belonging to G. jasminoides (BACH151).

** The sample was botanically described as belonging to G. florida (BACH152).

*DIH_2_O, deionized water*.

**Figure 1:**
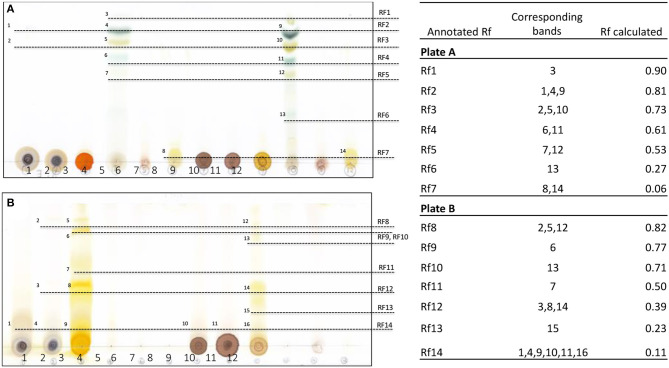
Thin-layer chromatography and calculated retention factor (Rf) values of separated bands of *Gardenia* extracts run on different mobile phase systems, namely benzene–ethyl acetate **(A)** and chloroform–methanol **(B)**; 1–3 are methanol extracts, 4–6 are ethyl acetate extracts, 7–9 are DIH_2_O extracts, and 10–12 are dichloromethane extracts of the leaves and flowers of *G. jasminoides* (BACH151) and fruit of *G. florida* (BACH152), respectively.

TLC is the technique used for separation of pigments, including chlorophylls and carotenoids, from plants and other biological substances (34, 35). Different types of extracts were run on the chromatogram plates using hydrophobic mobile-phase systems of different polarity. In our results, it is apparent that chlorophylls from *Gardenia* leaf extracted with non-polar solvents, namely ethyl acetate (Figure 1A, band 4) and dichloromethane (Figure 1A, band 10), are well-separated in the non-polar mobile phase (benzene–ethyl acetate). The result was in agreement with Katayama et al. (36), who demonstrated that hydrophobic solvents should be used to eliminate water contamination that could prevent clear chromatographic separation of the pigments. Based upon the power of solubility in the mobile phase, a retention factor (Rf) of 1–5 could represent different photosynthesis pigments, including pheophytins a and b, chlorophylls a and b, and xanthophyll (37). In another system, yellow pigments were separated well using methanol and DIH_2_O extracts of *Gardenia* fruit (Figure 1B, bands 5–9 and 12–16). Also, bands of flower and leaf samples in the same extracting solvents gave the same Rf values as those from fruit (R_f_ 8, 12, and 14). In a similar study, six bands representing yellow pigments were isolated from *Gardenia* fruits using high polar solvents (H_2_O and methanol), which were later identified as crocin and crocetin of the carotenoid groups (16, 38).

To further characterize the pigments, methanol and DIH_2_O extracts of leaf, flower, and fruit and ethyl acetate extracts of fruit were chosen as they showed some trace evidence of yellow pigment by observation and TLC separation.

### Ultraviolet and Nuclear Magnetic Resonance Spectra

**Figure 2:**
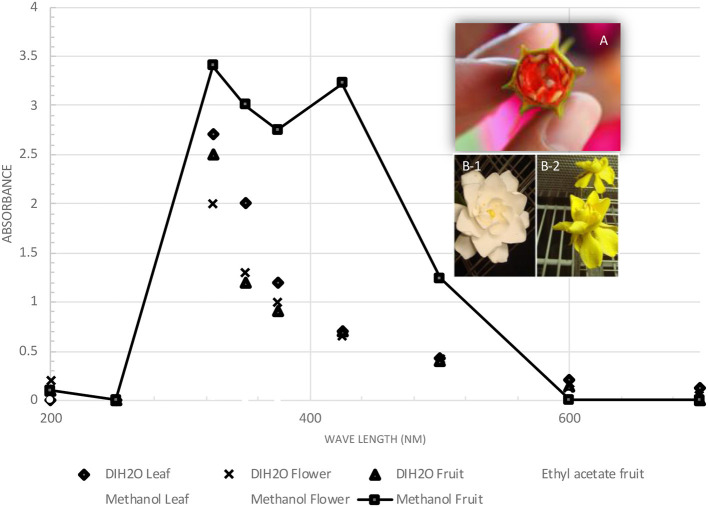
Ultraviolet spectra (200–700 nm) of *Gardenia* yellow extracts from different parts of *Gardenia* spp. **(A)** *Gardenia* fructus; **(B)** flowers of *Gardenia jasminoides* (BACH151).

The UV spectra of dye extracts from leaves, flowers, and fruits of *Gardenia* obtained by water and methanol extraction are illustrated in Figure 2. There was a single peak at 325 nm for the *Gardenia* fruit and flower extracts and two obvious peaks at 325 and 440 nm for ethyl acetate extracts of *Gardenia* fruits. The yellow pigments of the carotenoids usually give the maximum absorbance between 420 and 470 nm. The maximum absorbance of pure crocetin is at 420 nm, while crocin and crocetin with saccharide moieties exhibit maximum wavelengths at about 440 nm (16, 25, 26). Moras et al. (39) showed that the *cis* form of crocetin could absorb another maximum wavelength at ~300 nm.

**Figure 3:**
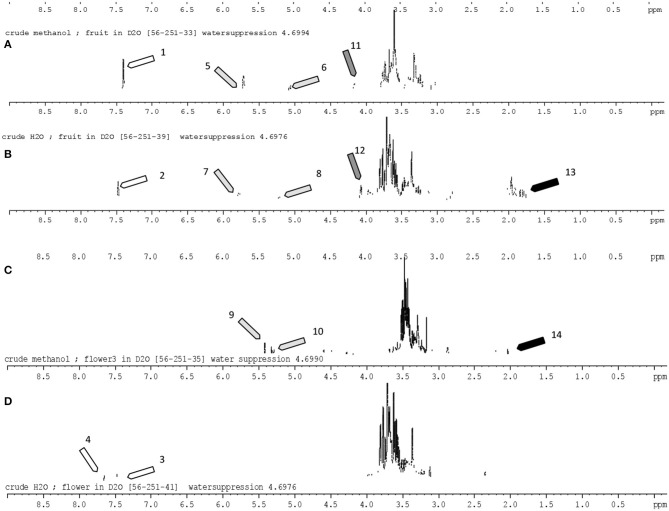
^1^H Nuclear magnetic resonance (300 mHz) spectrum of *Gardenia* fruit and flower extracts (in high polar solvents; DIH_2_O and methanol). The identified peaks corresponding to the peak number were in accord with the literature [Siwawej and Jarayapun (16); Choi et al. (22); Sobolev et al. (24); Srivastava et al. (40); Singh (41)]. Assignments: 1–3, *all-trans*-crocin; 4, 13-cis-crocin; 5,7, β-d-gentiobiosyl; 6,8, α-glucose; 9, DB (double bond); 10, CH-1 α-glucose; 11,12, xanthophylls; 13,14, CH3-19,20 crocetin. **(A,B)** Crude methanol and water extracts of *Gardenia* fruit; **(C,D)** crude methanol and water extracts of *Gardenia* flowers.

**Scheme 1:**
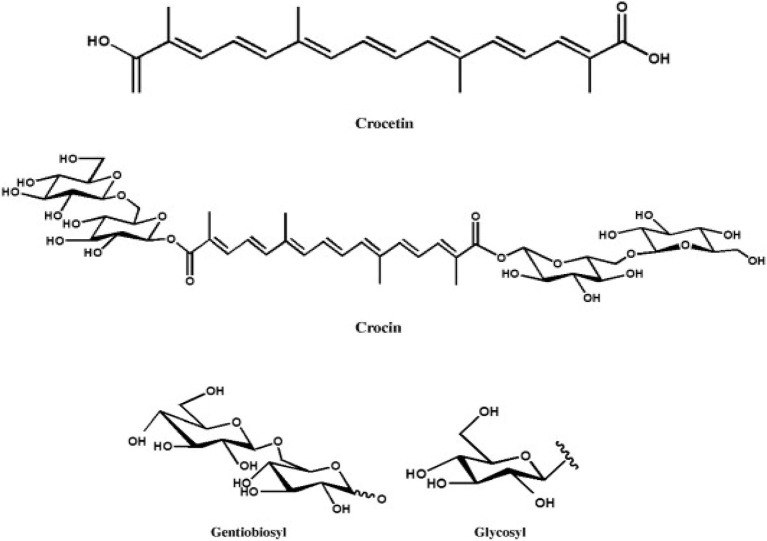
Crocetin and crocetin ester (crocin) with the aglycones.

As petals of mature *Gardenia* flower turned yellow over time (Figure 2B2), we anticipated that there should be some evidence of the yellow pigments in flower extracts and therefore structural elucidation by NMR was undertaken. Fruit and flower tissues extracted with methanol and DIH_2_O were scanned for 300 mHz ^1^H NMR spectrums (Figures 3A–D). By using the ^1^H NMR chemical shifts and integration signals (peaks) together, with data that have been reported from NMR spectroscopy of *Gardenia* pigments (16, 22, 24, 40, 41). The results illustrate that fruit and floral (DIH_2_O) extracts consisted of the major chemical shifts of CH-10 at 7.51 ppm and CH-10,10′ at 7.44 ppm of the *trans* form (peaks 1–4) as well as signals at ~2.0 ppm (peaks 13 and14) that are similar to those of crocetins (23, 38). Peaks 5–10 could also indicate aglycones (sugar moieties) such as β-d-gentiobiosyl, β- d -glucosyl, and α-glucose with the obvious chemical shifts of CH-1 at ~5.5 ppm and CH2-6′,6′ at 3.7 ppm (24). This confirms that the chemical structures of *Gardenia* yellow comprise crocetin esters, including, for example, crocetin digentiobioside ester (crocin), crocetin monogentiobioside monoglucoside ester, and crocetin diglucoside ester (22). Peaks 11 and 12 (chemical shifts at 4.2 ppm) together with peak 13 (signal at 2.1 ppm) with CH resonances at 3.21–3.23, 3.26–3.28, 3.23–3.25, and 3.17–3.19 ppm and CH-2 resonances at 3.67 and 3.45–3.47 ppm also indicate the existing xanthophylls in *Gardenia* fruit extract (41). The chemical structures of crocetin and its ester formed with aglycones (gentiobiosyl and glycosyl) are illustrated in Scheme 1.

### Capillary Electrophoresis–Mass Spectrometry

**Figure 4:**
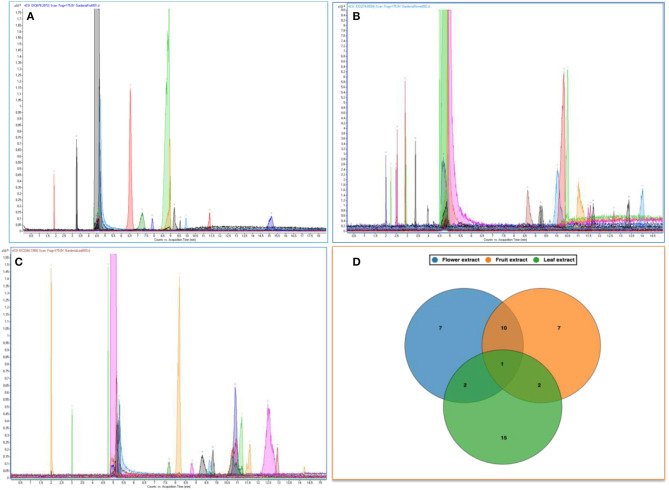
Electropherograms of methanolic extracts of *Gardenia* spp. fruit **(A)**, flower **(B)**, and leaf **(C)**. Venn diagrams of the common metabolites found in those extracts **(D)**.

**Figure 5:**
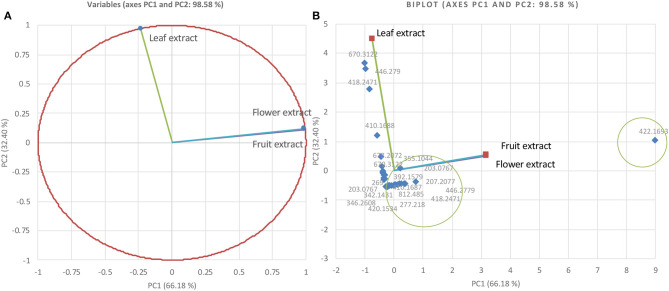
Principal component analysis relationship of mass values (*m*/*z*; positive ions) of the 20 most abundant components from *Gardenia* methanolic extracts **(A)** and biplot cluster analysis **(B)**.

The methanolic extracts of the *Gardenia* parts were first run with an aqueous buffer system to enable the analyses of cations and anions in a single run. This allowed us to separate or detect all compounds in the positive detection mode. Figures 4A–C illustrates the complex electropherograms of methanolic extracts of different parts of *Gardenia*. The Venn diagram illustrates that the extracts of floral and fruit tissues share 11 metabolites in common (Figure 4D). As it was impossible to identify such an intricate matrix, the 20 most abundant analytes for each extract were obtained with their corresponding *m*/*z*-values and peak area. As nearly all of the analytes are singly charged, the denoted *m*/*z*-values correspond to the mass of the pseudomolecular ion (positive ion). The relationship between the compounds analyzed from the methanolic extracts was evaluated in the form of mass trace PCA, as shown in Figure 5. In agreement with the Venn diagram, PCA illustrates the similarity of the compounds' patterns in the fruit and flower extracts leading to 11 compounds with a positive ion charge, as shown in Table 3. The crocins (crocetin and crocin 2) and the iridoids such as genipin gentiobioside and 8-epiapodantheroside were among the major components found in both *Gardenia* fruit and flower. Generally, the crocins are able to absorb at 430 nm. The other miscellaneous components, including jasminodiol, genistein, and gardenoside, absorb the maximum wavelength at ~240 nm. In other studies, numerous bioactive ingredients were isolated and identified from fruit of *Gardenia*, including the iridoids (42, 43), which show health benefits such as anti-inflammation, anti-depression, and anti-diabetic properties, anti-thrombotic activity, protection against lipopolysaccharide-induced apoptotic liver damage, and a proven antagonistic effect against Alzheimer's disease (43–45). Crocins are the glycosides (monoglycosyls or diglycosylesters) of C-20 carotenoid aglycone crocetins. They are also known to be the major compounds responsible for the unique yellow color of *Gardenia* and saffron (17, 22, 24). The other components isolated from fruits with bioactive functionality include the flavonoids, diterpenoids, and triterpenoids (46). A few studies have highlighted the isolation and characterization of phytochemical ingredients in the flower tissues of *Gardenia*, such as iridoids and triterpenoids (45, 47–49). In other studies the phenylpropanoid glucoside along with phenolic acids and flavones were isolated from the edible floral tissue of *G*. *jasminoides* and Zhizhi (9, 50). These compounds possess high antioxidant potential and elucidated neuroprotective, hepatoprotective, anti-inflammatory, antitumor, hypoglycemic, and hypolipidemic activities (51, 52). Thus, both the fruits and flowers of *Gardenia* spp. have the potential to be developed into medicinal food and pharmaceutical products (9). These studies, however, did not confirm the presence of crocins in the flower, in particular when the flower reached the mature stage. This paper is, therefore, the first evidence that the yellow dye crocins detected in the flower of *Gardenia* could be useful for finding alternative sources of natural food additives in a sustainable manner.

**Table 3:** Principal trace compounds identified from methanolic extracts of fruit and flower of *Gardenia* spp.

**Compound number**	**Formula**	**Positive ion**	**Other fragments**	**Compound identified**	**UV max. (nm)**	**Category**
1	C_10_H_16_O_3_	203.0767^[M+Na]+^	109, 123, 151, 139	Jasminodiol	242	Miscellaneous
2	C_15_H_10_O_5_	266.1613	225, 244	Genistein	240	Miscellaneous
3	C_17_H_24_O_11_	277.218	233.15, 247	Gardenoside	240	Miscellaneous
4	C_16_H_18_O_9_	355.1044^[M+Na]+^	135, 191	Crocetin	438	Crocins
5	C_16_H_20_O_10_	392.1579	300	Deacetyl-asperulosidic acid	234	Iridoids
6	C_23_H_34_O_15_	410.1687^[M+Na]+^	210	Genipin gentiobioside	240	Iridoids
7	C_16_H_24_O_11_	418.2471	177	Shanzhiside	240	Iridoids
8	C_17_H_24_O_10_	420.1534	–	8-epiapodantheroside	240	Iridoids
9	–	422.1693	–	Unknown		
10	–	446.2779	–	Unknown		
11	C_38_H_54_O_19_	812.485	651, 327, 283	Crocin 2	438	Crocins

## Conclusion

In this study, crocin 2 and crocetin were characterized for the first time from methanol and DIH_2_O extracts of fully mature flowers of *G. jasminoides*. They were structurally confirmed by means of NMR patterns and MS after separation by capillary electrophoresis in comparison with those of *Gardenia* yellow from Zhizhi. The other compounds were iridoids and triterpenoids, which have beneficial bioactivities that could be potential used as food additives and medicinally functional ingredients. In this sense, cape jasmine, a cultural ornamental plant, provides an opportunity to develop sustainable edible landscapes. The research information from this study could also increase the market value of the over-mature flowers that are often discarded.

## Data Availability Statement

The datasets generated for this study are available on request to the corresponding author.

## Author Contributions

The project idea and experimental design was developed and data were analyzed by SS. Sample collections were performed and the manuscript was written by SS and PS. The laboratory experiments were run by SS, KS, WR, and TJ. All authors contributed to the article and approved the submitted version.

## Conflict of Interest

The authors declare that the research was conducted in the absence of any commercial or financial relationships that could be construed as a potential conflict of interest.

## Botanical glossary

***Gardenia florida* L**. (syn. *Gardenia jasminoides* var. jasminoides*)*

An evergreen gardening shrub of the family Rubiaceae, bearing white fragrant flowers with a simple corolla. The flower is fertile and the fruit is ellipsoid to ovoid, orange when ripe with fleshy placentae and numerous seeds.

***Gardenia jasminoides*** **Ellis**

A common tropical landscape plant. It is quite variable morphologically especially in leaf size, calyx lobe size, and corolla size. Several varieties have been recognized but, in this work, the corolla is white to pale yellow and doubled that of var. fortuneana. The flower is sterile and the plant does not set fruit.

**Zhizhi or fructus**

Fruits of *Gardenia florida* L.
